# Resistance Patterns of Gram-Negative Bacteria Recovered from Clinical Specimens of Intensive Care Patients

**DOI:** 10.3390/microorganisms9112246

**Published:** 2021-10-28

**Authors:** Farooq Ahmed Wani, Altaf Bandy, Mohammed Jayed S. Alenzi, Abdulaziz Ibrahim Alzarea, Abdullah S. Alanazi, Mohammed Ubaidullah Sayeed, Ashokkumar Thirunavukkarasu, Bilal Tantry, Mushtaq Dar

**Affiliations:** 1 Department of Pathology, College of Medicine, Jouf University, Sakaka 72388, Saudi Arabia; usmohammed@ju.edu.sa; 2 Department of Community Medicine, College of Medicine, Jouf University, Sakaka 72388, Saudi Arabia; drbanday@gmail.com (A.B.); ashokkumar@ju.edu.sa (A.T.); 3 Department of Surgery, College of Medicine, Jouf University, Sakaka 72388, Saudi Arabia; mja@ju.edu.sa; 4 Department of Clinical Pharmacy, College of Pharmacy, Jouf University, Sakaka 72388, Saudi Arabia; aizarea@ju.edu.sa (A.I.A.); Asdalananzi@ju.edu.sa (A.S.A.); 5 Health Sciences Research Unit, Jouf University, Sakaka 72388, Saudi Arabia; 6 Department of Microbiology, Government Medical College, Kashmir University, Srinagar 190006, India; bilaltantry@gmail.com; 7 Ministry of Interiors, Jeddah 21577, Saudi Arabia; mushtaq164364@gmail.com

**Keywords:** intensive care units, Gram-negative bacteria, antimicrobial resistance, multidrug resistance, carbapenems

## Abstract

Intensive care units are complex environments favoring high resistance in microorganisms. This study evaluated the resistance and the distribution dynamics of resistant Gram-negative bacteria (GNB) in patients admitted to intensive care units. This retrospective, record-based, cross-sectional study analyzed all of the antibiograms of patients admitted to the ICUs. The BD Phoenix system (BD Diagnostics, Sparks, MD, USA) was used for bacterial identification and antimicrobial testing. Clinical and Laboratory Standard Institute recommendations were used for antimicrobial testing. Frequencies and percentages of multidrug and pan-drug resistance were calculated. A total of 570 bacterial growths were observed, out of which 437 (76.7%) were of GNB. *K. pneumoniae* (21.0%), *P. aeruginosa* (11.8%), and *Staphylococcus aureus* (13.2%) were the most frequent disease-causing bacteria in intensive care patients. Resistance rates of 73.2% and 70.1% were observed for third- and fourth-generation cephalosporins, respectively, while 48.2% carbapenem and > 65% fluoroquinolones resistance rates were observed. Amikacin was the most effective antibiotic, with a sensitivity rate of 69.5%. A total of 372 (85.1%) of GNB were multidrug resistant. The majority of infections in intensive care patients are caused by multidrug-resistant (MDR) Gram-negative bacteria. Female gender and advancing age are factors favoring MDR. Enhanced surveillance and strengthening of the antimicrobial stewardship program are warranted.

## 1. Introduction

Antimicrobial resistance is being increasingly recognized throughout the world, and is offsetting the success story of antibiotics. The world is encountering a grave threat of resistant bacterial infections, which have emerged as a significant public health problem. Microorganisms are developing new resistance mechanisms that increase their capacity to spread globally and cause prolonged illness, disability, and death. In the absence of efficacious antimicrobials, medical procedures are becoming increasingly risky, and result in more extended hospital stays, often requiring intensive care [1]. Although emanation of antimicrobial resistance is considered to be a natural phenomenon, it may be driven by the use of antimicrobial agents in healthcare settings, agriculture, and the environment. The drivers of increased antimicrobial resistance may include lack of optimal usage of antimicrobials, environmental contamination with antimicrobials, poor infection control measures, lack of sanitation and restricted access to clean water, increased international travel, and migration [2].

The World Health Organization (WHO) has published a list of antibiotic-resistant “priority pathogens” that includes 12 families of bacteria that constitute the greatest threat to human health. The list is divided into critical-, high-, and medium-priority pathogens. The critical group includes Gram-negative multidrug-resistant bacteria (Enterobacterales such as *K. pneumoniae*, *E. coli*, *S. marcescens*, *and P. mirabilis*; *A. baumannii*, *and P. aeruginosa*). These Gram-negative bacteria (GNB) have acquired resistance to the best accessible antibiotics, including carbapenems and cephalosporins [3]. These multidrug-resistant (MDR) organisms have been reported throughout the globe, posing great therapeutic challenges and resulting in increased morbidity and mortality [4].

Health care-associated infections (HCAIs) are being reported increasingly in developed and developing countries. The WHO reports a pooled prevalence of HCAIs of 7.6% in mixed patient populations in high-income countries (1995–2010), ranging from as low as 3.6% in Germany to as high as 12% in New Zealand. The same WHO report observed pooled prevalence of HCAIs of 15.5% in mixed patient populations in low- and middle-income countries (1995–2010), ranging from 5.4% in Mongolia to 19.1% in Albania [5]. The EPIC II study noted that 51% of the patients admitted to the ICUs were infected, thereby indicating infection as a significant reason for increased morbidity and mortality in the ICUs [6] Patients admitted to the ICUs are particularly prone to developing infections because of numerous risk factors, including the presence of comorbid conditions, central venous catheterization, mechanical ventilation, urinary catheterization, usage of steroid and antibiotics, and lengthier ICU stays [7]. Infections acquired in ICUs are an independent risk factor for increased hospital mortality [8].

Although many studies have been carried out in Saudi Arabia [9,10,11,12] regarding GNB in ICU settings, studies from the northern region are scarce. The present study was planned keeping in view the necessity of having regional data on antimicrobial resistance that can be used for planning and implementing the antimicrobial stewardship programs so as to improve the clinical outcomes of the patients admitted to the intensive care units. We evaluated the resistance patterns—especially the MDR patterns—and the distribution dynamics of the resistant Gram-negative bacteria in patients admitted to intensive care units.

## 2. Materials and Methods

### 2.1. Study Design

The study was conducted in a referral hospital situated in the city of Sakaka in the Al-Jouf region for a period of one year, and was completed by December 2020. This region lies in the north of Saudi Arabia. This study followed a single-center, record-based, cross-sectional design. The research project was approved by the Jouf University’s Local Committee on Bio-Ethics via research protocol no. 03/04/41 dated 6 January 2020. As our study involved the analysis of antibiograms from the microbiology unit of the study hospital, there was no direct contact with the patients; hence, no consent was required. However, as a standard operating procedure practiced in this hospital, patients’ and/or relatives’ consent is taken before carrying out any procedure on patients admitted to ICUs, and the same is recorded in the files.

### 2.2. Bacterial Identification and Antimicrobial Susceptibility Testing

The BD Phoenix system (BD Diagnostics, Sparks, MD, USA) was used for bacterial identification and testing. Clinical and Laboratory Standard Institute guidelines were used for antimicrobial testing and its interpretation [13]. Distribution data of clinical specimens, along with patient demographics, were also retrieved. The resistance patterns exhibited by the bacteria were based on an earlier classification by Magiorakos et al. [14].

The antibiotic panel consisted of amikacin (AMK), gentamicin (GEN), ertapenem (ETP), imipenem (IPM), meropenem (MEM), cephalothin (CEF), cefuroxime (axetil or sodium) (CXM), cefoxitin (FOX), ceftazidime (CAZ), ceftriaxone (CRO), cefepime (FEP), aztreonam (ATM), ampicillin (AMP), amoxicillin/clavulanic acid (AMC), piperacillin/tazobactam (TZP), colistin (CST), trimethoprim/sulfamethoxazole (SXT), nitrofurantoin (NIT), ciprofloxacin (CIP), levofloxacin (LVX), and tigecycline (TGC).

### 2.3. Statistical Analysis

Data on the antimicrobial test results were coded before entry. The correctness of data entry was ensured independently by researchers. Intermediate resistance exhibited by the microorganisms was considered resistant for this study. Data were analyzed using Statistical Package for the Social Sciences (SPSS) version 22.0 for Windows (IBM SPSS Statistics, IBM Corporation, Armonk, NY, USA). Frequencies and percentages of MDR, extensive-drug resistant (XDR), Pan-drug resistant (PDR), and their distribution in clinical samples and different age groups were calculated.

## 3. Results

Of the 570 bacterial growths, 437 (76.7%) were Gram-negative, and 133 (27.3%) were Gram-positive isolations. A total of 313 (55.0%) of these infections occurred in males, and 199 (35.0%) in patients above 75 years of age. *K. pneumoniae* (21.0), *P. aeruginosa* (11.8%), and *Staphylococcus aureus (13.2%)* were the most common bacteria causing infections in intensive care patients. Bacteria were frequently isolated from blood (28.8%) and urine (23.5%) specimens. Almost half of the infections (250; 43.9%) occurred in the first quarter (January to March) of the study period (Table 1).

**Figure 1:**
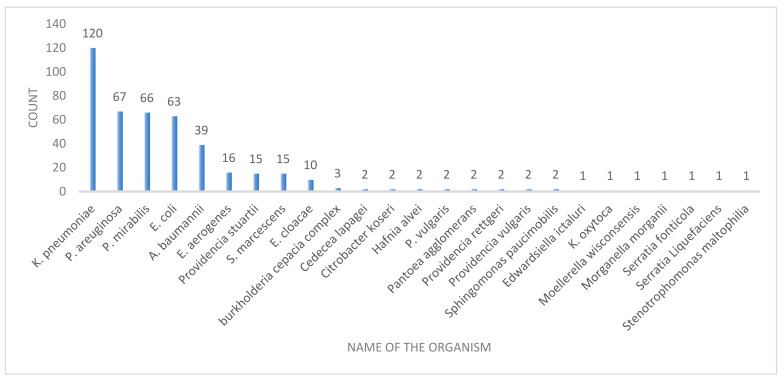
Gram-negative bacteria isolated from ICU patients.

Of the 437 Gram-negative infections, 221 (50.6%) occurred in males, and 141 (32.3%) in patients aged more than 75 years. Nearly half (47.7%) of the bacterial isolations were recovered in the first quarter. Urine specimens (131; 30.0%) dominated the frequency of Gram-negative bacterial growths, followed by blood specimens (112; 25.6%). The majority of the infections were caused by *K. pneumoniae* (27.5%) (Table 2 and Figure 1).

**Figure 2:**
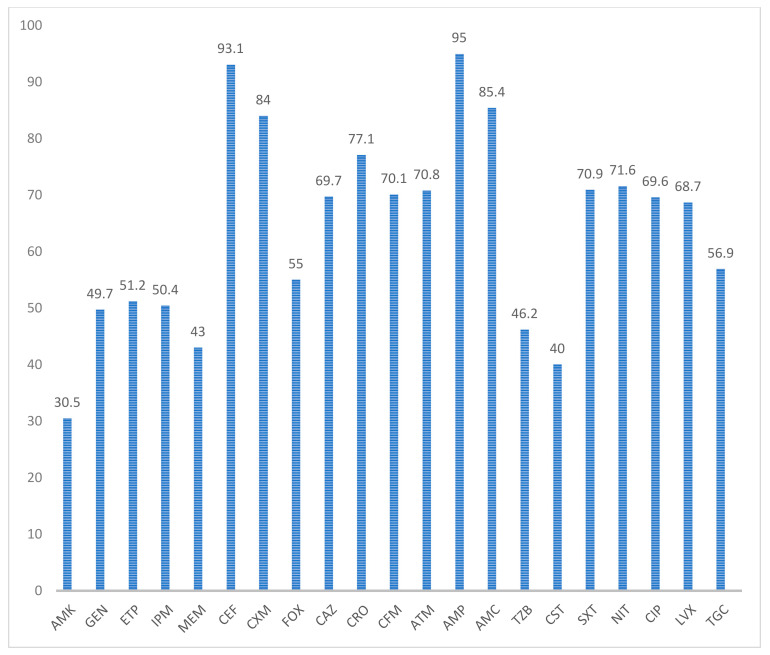
Antimicrobial resistance exhibited by Gram-negative organisms: amikacin (AMK), gentamicin (GEN), ertapenem (ETP), imipenem (IPM), meropenem (MEM), cephalothin (CEF), cefuroxime (axetil or sodium) (CXM), cefoxitin (FOX), ceftazidime (CAZ), ceftriaxone (CRO), cefepime (FEP), aztreonam (ATM), ampicillin (AMP), amoxicillin/clavulanic acid (AMC), piperacillin/tazobactam (TZP), colistin (CST), trimethoprim/sulfamethoxazole (SXT), nitrofurantoin (NIT), ciprofloxacin (CIP), levofloxacin (LVX), tigecycline (TGC).

The highest antimicrobial resistance was observed against ampicillin (95%) and cephalothin (93.1%). An overall 73.2% and 70.1% resistance was observed for third- and fourth-generation cephalosporins. An overall carbapenem resistance rate of 48.2% was observed, with the lowest for meropenem (43%). A resistance rate of >65% was observed for fluoroquinolones. Amikacin was the most effective antibiotic, with a sensitivity rate of 69.5% (Figure 2).

Overall, 45 (10.3%) were resistant to less than three antibiotic classes, while 372 (85.1%) were multidrug resistant, which also included 125 (28.6%) of the extensively drug-resistant and 20 (4.6%) of the pan-drug-resistant isolates of the Gram-negative bacteria. All isolates of *A. baumannii* were multidrug-resistant strains, and all isolates of the *Burkholderia cepacia complex* were pan-drug resistant. Among the frequently isolated Gram-negative bacteria, 60 (95.2%) of *P. aeruginosa*, 57 (86.4%) of *P. mirabilis*, 100 (83.3%) of a *K. pneumoniae*, and 50 (79.4%) of *E. coli* were multidrug resistant (Table 3).

As regards the multidrug-resistant strains, 88.9% were recovered from female patients and 81.45% from male patients admitted to intensive care units. MDR isolation rates of around 90% were observed for the blood, tracheal wash, and sputum samples. MDR isolates were also recovered at a higher frequency (>75%) from all age groups (Table 4).

## 4. Discussion

ICUs are sections of hospitals with a complex environment where multidrug-resistant organisms are maximal, posing significant difficulties in the treatment of patients [15]. The problem of MDR in ICU settings is diversified and multifold, as ICUs cater to critically ill patients afflicted with life-threatening conditions and infected with a wide variety of infectious agents [16]. The complexity of the ICU environment favors the acquisition and spread of nosocomial infections. ICU patients are increasingly subjected to invasive procedures such as central venous catheterization, arterial catheterization, intra-aortic balloon pump (IABP), tracheostomy, etc. They may have multiple indwelling devices such as urinary catheters, feeding tubes, etc., which serve as a continuous source of infectious agents and a potential point of entry for infectious organisms. Furthermore, the involvement of multiple healthcare professionals creates a desirable environment for the person-to-person transmission of infectious diseases. In addition, unwarranted use of broad-spectrum antimicrobials may favor the emergence of infectious strains [17].

The present study was planned keeping in view the grave dangers posed by the MDR bacteria in the ICUs. It is critical to assess the resistance patterns of the MDR bacteria and determine the factors involved in the acquisition and dissemination of these bacteria. This will be essential for planning proper treatment protocols for managing infections in ICUs.

The majority of infections occurred in the first quarter of the year (47.4%), which could be explained by the fact that infection rates are higher in the winter months because of many factors, such as crowding of people at home, inadequate ventilation, low vitamin D levels, dry air, and decreased physical activity [18]. We observed that the majority (76.7%) of the isolates were Gram-negative, and only 27.3% were Gram-positive isolations. A total of 55.0% of the infections were noted in males, and 35.0% of the infections occurred in patients above 75 years of age (Table 1). Gram-negative infections were more frequently observed in males (50.6%) and in patients aged more than 75 years (32.3%) (Table 2). Ibrahim observed in 2018 that 81% of the isolates were Gram-negative, and male patients (59.7%) were predominant in that study [9]. The predominance of Gram-negative isolates in older male patients admitted to ICUs has been observed in studies conducted by Bianco et al., Sligl et al., and Lachhab et al. [19,20,21]. Males are generally more prone to infections than females, due to differences in the sex steroids as well as imbalance of sex-chromosome-linked genes [22]. The increased incidence in older age groups may be due to their defective defense mechanisms and deteriorating immune systems [21,23].

The most common bacteria detected in the ICUs in our study were *K. pneumoniae* (21.0), *P. aeruginosa* (11.8%), and *Staphylococcus aureus* (13.2%) (Table 1). Iwuafor et al. found *K. pneumoniae* (13.6%) to be the most common Gram-negative organism; bloodstream infections (49%), followed by urinary tract infections (35.6%), were the most common infections in their study [24] *K. pneumoniae* was the predominant organism noted in the studies carried out by Oliviera et al., Pokhrel et al., and Wang et al. [25,26,27]. *K. pneumoniae* is a facultative anaerobe, and is a common pathogen in hospital settings. Because of unrestricted use of broad-spectrum antimicrobials, and selective pressure applied therein, there is an increased generation of MDR and carbapenem-resistant strains of *K. pneumoniae* [28]. The predominance of bloodstream infections has been noted in studies carried out by Ak O et al., Moremi et al., and Frattari et al. [29,30,31]. In our study, Gram-negative isolates were recovered mainly from urine specimens (30.0%), followed by blood specimens (25.6%) (Table 2). Maximum isolations from urinary samples were observed by Ozer et al. and Mythri et al. [7,23].

The highest antimicrobial resistance was observed against ampicillin (95%) and cephalothin (93.1%) (Figure 2). Kumari et al. observed the highest resistance against ampicillin (97.6%) and first-generation cephalosporins (98.8%) [32]. An overall 73.2% and 70.1% resistance was observed for third- and fourth-generation cephalosporins in our study (Figure 2). Ibrahim and Tan et al. also observed higher resistance rates, ranging from 63.3 to 70% and 75 to 88%, respectively, against third- and fourth-generation cephalosporins [9,33]. A > 65% resistance rate was observed for fluoroquinolones in our study (Figure 2). A 61.5% resistance rate for ciprofloxacin was observed by Ibrahim [9]. Tan et al. noted resistance of >72% for fluoroquinolones [33].

We observed an overall carbapenem resistance rate of 48.2%, with the lowest for meropenem (43%) (Figure 2). Moolchandani et al. observed resistance of 33.9% in their study [34], while Ibrahim noted a resistance rate of >53% against carbapenems [9].

Amikacin was the most effective antibiotic, with a sensitivity rate of 69.5% in our study (Figure 2). Kumari et al. also observed the lowest mean resistance for amikacin (48.5%), making it the most effective drug in their study [32]. Amikacin has also been found to be an effective drug in other studies; resistance rates of 48.1%, 40.5%, and 43% were observed by Ibrahim, Moolchandani et al., and Leelarasamee et al., respectively [9,34,35].

Multidrug resistance (MDR) was observed in 372 (85.1%) isolates of the Gram-negative bacteria (Table 3). An increased frequency of MDR isolates represents a worrying trend in treating the patients in the ICUs, warranting strict infection control measures in the hospitals and judicious use of antimicrobials. Similarly high degrees of MDR isolates (87.07% and 89.5%) were also seen in the studies conducted by Siwakoti et al. [36] and Agyepong et al. [37], respectively. Ibrahim [9] noted MDR in 67.9% of isolates, whereas Moolchandani et al. [34] observed MDR in 55.7% of isolates.

All isolates of *A. baumannii* were MDR, whereas MDR isolates were seen in 60 (95.2%) of *P. aeruginosa*, 57 (86.4%) of *P. mirabilis*, 100 (83.3%) of *K. pneumoniae*, and 50 (79.4%) of *E. coli* (Table 3). Bianco et al. noted MDR phenotypes in all *P. aeruginosa*, and in 91.6% of *A. baumannii*, 40% of *E. coli*, and 52.3% of *K. pneumoniae* isolates [19], Ibrahim observed MDR in 97.5% of *A. baumannii*, 60.9% of *P. aeruginosa*, 46.7% of *P. mirabilis*, 59.3% of *K. pneumoniae*, and 43.2% of *E. coli* isolates [9]. Higher degrees of resistance observed in *A. baumannii* and *P. aeruginosa* have been attributed to the intrinsic resistance of these organisms to antimicrobials, as well as their potential to acquire resistant genes. Multiple resistance mechanisms include production of β-lactamases, metallo-carbapenemases, and aminoglycoside-modifying enzymes, as well as structural modifications in the membrane, mutations in topoisomerases, and increased activity of efflux pumps [38].

Overall, we observed 125 (28.6%) extensively drug-resistant isolates of the Gram-negative bacteria (Table 3). XDR was noted in 79.5% of *A. baumannii*, 38.8% of *P. aeruginosa*, 9.09% of *P. mirabilis*, 21.7% of *K. pneumoniae*, and 20.6% of *E. coli* isolates. Hasanin et al. noted XDR in 65% of Gram-negative isolates, with *A. baumannii* (86%), *P. aeruginosa* (63%), *P. mirabilis* (61%), *K. pneumoniae* (52%), and *E. coli* (47%) [39]. Parajuli et al. noted XDR in 43.3% of Gram-negative isolates, with *Acinetobacter* spp. (84.4%), *Pseudomonas* spp. (62.5%), *Klebsiella* spp. (48.6%), and *E. coli* (19.3%) [16]. The higher rates observed by Hasanin et al. and Parajuli et al. may be due to the local factors prevalent in their settings, such as unlimited antimicrobial use, increased induction, and dissemination of resistant strains. Many other studies, such as those conducted by Souza et al. and Qin et al., have reported lower XDR isolates in their studies [40,41].

Multidrug-resistant strains (88.9%) were recovered more frequently from female patients admitted to intensive care units. MDR isolates were also recovered at a higher frequency (>75%) from all age groups (Table 4). However, the predominance of MDR isolation in male patients has been observed by most other authors, including Ibrahim [9], Phu et al. [42], and Wang et al. [43]. The higher rates MDR isolates in female patients seen in our study may be attributed to the fact that rates of female hospital admission were almost equal to the rates of male admission, whereas in the studies cited above the rates of male hospital admission far exceeded the female admission rates.

This is the first study on antimicrobial resistance observed in various clinical specimens of patients admitted to this region’s intensive care units. This study highlights the importance of identifying the reasons for this high antimicrobial resistance, and developing strategies to tackle this issue. The use of recent data on antimicrobial resistance is the major strength of this study. The non-availability of molecular data on this resistance is the main limitation of this study.

## 5. Conclusions

We conclude that Gram-negative bacteria cause the majority of infections in intensive care patients. A high frequency of MDR Gram-negative bacteria was observed, with moderate amounts of XDR bacteria. Factors favoring MDR include female gender and advancing age.

The increased frequency of MDR Gram-negative bacteria represents a worrying trend, and warrants enhanced surveillance programs, strict infection control measures, and strengthening of the antimicrobial stewardship program.

## Figures and Tables

**Table 1:** Bacteriological diversity and distribution of ICU pathogens (*n* = 570).

Category		Number (*n*)	Percentage (%)
Gram-negative bacteria [437 (76.7%)]	*K. pneumoniae*	120	21.0
	*P. aeruginosa*	67	11.8
	*P. mirabilis*	66	11.6
	*E. coli*	63	11.0
	*A. baumannii*	39	6.8
	*E. aerogenes*	16	2.8
	*Providencia stuartii*	15	2.6
	*S. marcescens*	15	2.6
	Others	36	6.3
Gram-positive bacteria [133 (23.3%)]	*Staphylococcus aureus*	75	13.2
	*Enterococcus faecalis*	12	2.1
	*Streptococcus* spp.	19	3.3
	*Staphylococcus capitis*	5	0.9
	*Staphylococcus haemolyticus*	4	0.7
	*Staphylococcus epidermidis*	3	0.5
	Others	15	2.6
Gender	Males	313	55.0
	Females	257	45.0
Quarter	Quarter 1	250	43.9
	Quarter 2	136	23.9
	Quarter 3	76	13.3
	Quarter 4	108	19.0
Age (in years)	>75	199	35.0
	61–75	118	20.7
	46–60	92	16.1
	31–45	52	9.1
	16–30	69	12.1
	≤15	40	7.0
Type of specimen	Blood	164	28.8
	Urine	134	23.5
	Wound swab	94	16.5
	Sputum	75	13.2
	Nasal swab	54	9.5
	Tracheal wash	23	4.0
	Conjunctival swab	18	3.2
	Others	8	1.4

**Table 2:** Distribution of Gram-negative bacteria isolated from ICU clinical specimens (*n* = 437).

Category		Number (*n*)	Percentage (%)
Gender	Males	221	50.6
	Females	216	49.4
Name of the Gram-negative Bacteria	*K. pneumoniae*	120	27.5
	*P. aeruginosa*	67	15.3
	*P. mirabilis*	66	15.1
	*E. coli*	63	14.4
	*A. baumannii*	39	8.9
	*E. aerogenes*	16	3.7
	*Providencia stuartii*	15	3.4
	*S. marcescens*	15	3.4
	Others	36	8.2
Quarter	Quarter 1	207	47.4
	Quarter 2	108	24.7
	Quarter 3	48	11.0
	Quarter 4	74	16.9
Age (in years)	>75	141	32.3
	61–75	95	21.7
	46–60	78	17.8
	31–45	38	8.7
	16–30	58	13.3
	≤15	27	6.2
Type of specimen	Urine	131	30.0
	Blood	112	25.6
	Wound swab	86	19.7
	Sputum	65	14.9
	Tracheal wash	20	4.6
	Conjunctival swab	17	3.9
	Others	6	1.4

**Table 3:** Numbers of Gram-negative isolates showing multidrug, extensive-, and pan-drug resistance.

Microorganism	<3 abs *	MDR ^	XDR ˜	PDR **
*K. pneumoniae* (*n* = 120)	14	100	26	6
*P. aeruginosa* (*n* = 67)	3	60	26	4
*P. mirabilis* (*n* = 66)	8	57	6	1
*E. coli* (*n* = 63)	13	50	13	0
*A. baumannii* (*n* = 39)	0	39	31	0
*E. aerogenes* (*n* = 16)	1	13	11	2
*S. marcescens* (*n* = 15)	1	12	0	2
*E. cloacae* (*n* = 10)	1	9	3	0
*Burkholderia cepacia complex* (*n* = 3)	0	0	0	3
*Cedecea lapagei* (*n* = 2)	0	2	1	0
*Citrobacter koseri* (*n* = 2)	0	2	0	0
*Hafnia alvei* (*n* = 2)	0	0	0	2
*P. vulgaris* (*n* = 2)	0	2	0	0
*Pantoea agglomerans* (*n* = 2)	0	2	0	0
*Providencia rettgeri* (*n* = 2)	0	2	1	0
*Providencia stuartii* (*n* = 2)	2	13	4	0
*Providencia vulgaris* (*n* = 2)	0	2	1	0
*Stenotrophomonas maltophilia* (*n* = 2)	0	1	1	0
*Edwardsiella ictaluri* (*n* = 1)	0	1	0	0
*K. oxytoca* (*n* = 1)	0	1	0	0
*Moellerella wisconsensis* (*n* = 1)	0	1	0	0
*Morganella morganii* (*n* = 1)	0	1	0	0
*Serratia fonticola* (*n* = 1)	0	1	1	0
*Serratia Liquefaciens* (*n* = 1)	0	1	0	0
*Sphingomonas paucimobilis* (*n* = 1)	2	0	0	0
Total (437)	45 (10.3%)	372 (85.1%)	125 (28.6%)	20 (4.6%)

* abs: antibiotics; ^ MDR: multidrug resistant; ˜ XDR: extensively drug resistant; and ** PDR: pan-drug resistant.

**Table 4:** Distribution of numbers of multidrug-, extensively drug-, and pan-drug-resistant isolates among different categories of patients.

Characteristic	<3 abs *	MDR ^	XDR ˜	PDR **
Gender				
Male (*n* = 221)	29	180	56	12
Female (*n* = 216)	16	192	69	8
Type of sample				
Urine (*n* = 131)	24	106	22	1
Blood (*n* = 112)	8	101	42	3
Wound swab (*n* = 86)	10	71	27	5
Sputum (*n* = 65)	0	59	19	6
Tracheal wash (*n* = 20)	1	18	8	1
Conjunctival swab (*n* = 17)	2	12	3	3
Others (*n* = 6)	0	5	4	1
Age (in years)				
>75 (*n* = 141)	12	122	39	7
61–75 (*n* = 95)	9	83	27	3
46–60 (*n* = 78)	8	68	17	2
31–45 (*n* = 38)	4	31	14	3
16–30 (*n* = 58)	11	45	19	2
≤15 (*n* = 27)	1	23	9	3

* abs: antibiotics; ^ MDR: multidrug resistant; ˜ XDR: extensively drug resistant; and ** PDR: pan-drug resistant.

## Data Availability

Data will be made available on request.
